# Optimizing Nutrition in Preterm Low Birth Weight Infants—Consensus Summary

**DOI:** 10.3389/fnut.2017.00020

**Published:** 2017-05-26

**Authors:** R. Kishore Kumar, Atul Singhal, Umesh Vaidya, Saswata Banerjee, Fahmina Anwar, Shashidhar Rao

**Affiliations:** ^1^Cloudnine Hospital, Bangalore, Karnataka, India; ^2^Institute of Child Health, UCL, London, United Kingdom; ^3^KEM Hospital, Pune, India; ^4^Nestle Nutrition, Kolkata, India; ^5^Medical and Scientific Affairs, Nestle Nutrition, South Asia Region, Gurgaon, India

**Keywords:** optimizing nutrition, preterm low birth weight infants, enteral feeding, expressed breast milk, donor pasteurized human milk, fortification

## Abstract

Preterm birth survivors are at a higher risk of growth and developmental disabilities compared to their term counterparts. Development of strategies to lower the complications of preterm birth forms the rising need of the hour. Appropriate nutrition is essential for the growth and development of preterm infants. Early administration of optimal nutrition to preterm birth survivors lowers the risk of adverse health outcomes and improves cognition in adulthood. A group of neonatologists, pediatricians, and nutrition experts convened to discuss and frame evidence-based recommendations for optimizing nutrition in preterm low birth weight (LBW) infants. The following were the primary recommendations of the panel: (1) enteral feeding is safe and may be preferred to parenteral nutrition due to the complications associated with the latter; however, parenteral nutrition may be a useful adjunct to enteral feeding in some critical cases; (2) early, fast, or continuous enteral feeding yields better outcomes compared to late, slow, or intermittent feeding, respectively; (3) routine use of nasogastric tubes is not advisable; (4) preterm infants can be fed while on ventilator or continuous positive airway pressure; (5) routine evaluation of gastric residuals and abdominal girth should be avoided; (6) expressed breast milk (EBM) is the first choice for feeding preterm infants due to its beneficial effects on cardiovascular, neurological, bone health, and growth outcomes; the second choice is donor pasteurized human milk; (7) EBM or donor milk may be fortified with human milk fortifiers, without increasing the osmolality of the milk, to meet the high protein requirements of preterm infants; (8) standard fortification is effective and safe but does not fulfill the high protein needs; (9) use of targeted and adjustable fortification, where possible, helps provide optimal nutrition; (10) optimizing weight gain in preterm infants prevents long-term cardiovascular complications; (11) checking for optimal weight and sucking/swallowing ability is essential prior to discharge of preterm infants; and (12) appropriate counseling and regular follow-up and monitoring after discharge will help achieve better long-term health outcomes. This consensus summary serves as a useful guide to clinicians in addressing the challenges and providing optimal nutrition to preterm LBW infants.

## Introduction

About 15 million preterm births are recorded each year worldwide. According to the World Health Organization, there has been an increase in the incidence of preterm births over the past 20 years in 62 of the 65 countries with available trend data. Over one million children die from preterm birth-related complications annually. Preterm births are the leading cause of newborn deaths and the second leading cause of death, after pneumonia, in children under the age of 5 years. Furthermore, survivors of preterm birth are at a higher risk of adverse developmental disabilities (1). They have higher rates of adverse health outcomes in early adulthood compared with their term counterparts (2). The various adverse developmental outcomes in adults born preterm have been outlined in Table 1 (3–9).

**Table 1:** **Adverse developmental sequelae in adults born preterm**.

Parameter	Adverse sequelae in adults born preterm
Neurological	Significant decrease in brain volume (3). Increased risk of neurological disabilities (4).
Cardiovascular and metabolic	Low insulin sensitivity and high blood pressure (5). Increased intra-abdominal fat and higher risk of metabolic complications (6). Increased arterial stiffness (7). Reduced ventricular size and volume; impaired systolic function (8).
Bone health	Significantly lower bone mineral density (9).
Others	Increased risk of social disabilities in adulthood (in terms of educational level attained, income, and establishment of family) (4).

Although there has been an improvement in the overall mortality in extremely premature infants in recent times, there is a rising need to develop newer strategies for lowering the potential complications of preterm birth (10).

### Nutrition—An Important Factor Influencing Developmental Outcomes in Preterm Infants

Nutrition is essential for growth, metabolism, and immunity in a preterm newborn low birth weight (LBW) infant (11–13). In a preterm infant, poor nutrition is associated with poorer head growth; persistent smaller head size results in poor psychomotor and mental skills, higher rates of cerebral palsy, and autism (14). Impaired weight and growth in preterm infants are significantly associated with adverse neurodevelopmental outcomes in later life (15). Barker’s hypothesis also states that infants with LBW are at a higher risk of coronary heart disease, hypertension, and type 2 diabetes in adulthood (16, 17).

## Objectives and Methods

Considering the significant contribution of nutrition to the development of preterm newborn LBW infants, a key opinion leader who has extensive expertise in the related domain reached out to other experts who had proficiency in various areas related to the topic/s proposed to be discussed during the consensus meeting. This was followed by the selection of a group of neonatologists, pediatricians, and nutrition experts, who convened in August 2016 to brainstorm and address the various challenges in providing optimal nutrition to preterm LBW infants. The panel discussion also focused at highlighting the advantages and disadvantages, optimal intakes, and practical recommendations for various enteral nutrition supplementation strategies.

This consensus summary paper compiles the evidence-based recommendations from the Expert panel and serves as a useful tool to the clinicians in optimizing nutrition in LBW preterm infants for beneficial health outcomes in the long term.

## Effects of Early and Aggressive Nutritional Strategies

It has been noted that better nutrition in the early postnatal phases in preterm infants results in higher verbal intelligence quotient (IQ) scores and improved cognitive function in the long term (18, 19). Higher protein and energy intake during the first week after birth in extremely LBW infants is associated with higher mental development index scores and lower risk of growth retardation at 18 months after birth (20). Early and higher protein and energy intake have also been correlated with faster head growth and an increase in head circumference in preterm infants (21, 22); increase in head circumference has been positively correlated with improved cognitive outcomes (23). Therefore, the administration of early aggressive nutritional enteral and parenteral support may help improve growth and developmental outcomes in preterm newborn LBW infants (24).

## Challenges in Providing Nutrition to Preterm Infants

### Parenteral versus Enteral Feeding

Enteral feeding is preferred to parenteral feeding, as the latter may be associated with catheter-related complications, infections, and sepsis, among others (25, 26). However, institution of early parenteral nutrition can sometimes be critical and a necessary adjunct to enteral therapy.

### Does Enteral Feeding Carry an Increased Risk of Necrotizing Enterocolitis (NEC) and Infections?

One of the most feared complications of enteral feeding of a preterm infant is the risk of NEC; this came into limelight based on a study published in *The Lancet* in 1990. In this study, NEC was more common in preterm infants fed formula milk compared to infants fed breast milk (27). However, there have been improvements in feeding regimens and methods since this publication. In studies published after this paper, there has been no increase in the risk of NEC with fast or early enteral feeding of expressed breast milk (EBM) or formula milk as compared to slow or delayed introduction of enteral feeding in LBW infants (28, 29). Furthermore, enteral feeding has been found to aid in the development of the gut and lower the risk of infections and sepsis (26, 30).

### When Should Enteral Feeding Be Started: Early or Late?

Delaying the administration of progressive enteral feeding to reduce the risk of NEC may not be an appropriate approach. Systematic reviews have reported that delaying enteral feeding does not lead to a reduction in the risk of NEC. On the contrary, this approach may prolong the time to achieve full enteral feeding (31). Furthermore, early versus late (<48 versus >72 h after birth) initiation of enteral feeding has been found to be associated with a significantly lesser time to gain birth weight, and shorter duration of parenteral nutrition and hospital stay, without any increase in the complication rate (32). A reduced incidence of osteopenia of prematurity and jaundice has also been noted with early versus late enteral feeding in very LBW infants (33).

### Advancement of Volume of Enteral Feed: Rapid versus Slow

Several studies have assessed the outcomes with rapid versus slow advancement of enteral feeding in preterm infants. In a study conducted in neonates with LBW (<1,250 g), rapid versus slow advancement of enteral feeding was associated with a significantly lesser time required to achieve full enteral feeding and regain birth weight; a shorter duration of hospital stay; comparable feed tolerance; and no increase in the risk of NEC. EBM was used in this study (28). Several other randomized studies and systematic reviews using formula milk have also reported similar findings (31, 34, 35).

### Frequency of Feeding: Continuous versus Hourly

Continuous nasogastric versus intermittent bolus milk feeding in preterm LBW infants has long been a topic of debate. While a few studies reveal comparable outcomes with the two feeding methods, a few others support a significantly faster growth rate and achievement of full enteral feeding with the continuous feeding technique (36, 37). A systematic review of these feeding methods in infants weighing less than 1,500 g reported faster weight gain and earlier hospital discharge with the continuous tube feeding method (38). Furthermore, a significant increase in pulmonary resistance, airflow, and respiratory instability and a decrease in cerebral perfusion have been noted with the bolus feeding method (39, 40) Therefore, continuous feeding seems to be logical in preterm infants till the development of sucking–swallowing coordination.

### How to Feed Enterally: Nasogastric versus Orogastric Route

The passage of nasogastric tube has been noted to increase airway resistance in preterm infants by 30–50% (41). An increased incidence of periodic breathing and central apnea has also been noted with nasogastric tubes, in preterm infants (42). Hence, the routine use of nasogastric tubes is not advisable in preterm infants.

### Can Preterm Infants Be Fed While on Ventilator or Continuous Positive Airway Pressure (CPAP)?

Ventilator or CPAP treatment should not serve as a hindrance to enteral feeding. Assisted ventilation does not increase the risk of gastroesophageal reflux and is, therefore, not a contraindication to enteral feeding in preterm LBW infants (43). Although nasal CPAP therapy results in gaseous bowel distension or the CPAP belly syndrome in the majority of very LBW infants, this may not be attributed to NEC; the feeding method has no correlation with the occurrence of the CPAP belly syndrome (44).

### Is Routine Evaluation of Gastric Aspiration and Abdominal Girth Justified?

Gastric aspiration and evaluation of gastric residuals may delay enteral tube feeding and cause damage to the gastric mucosa (45, 46). Increase in abdominal girth < 1.5 cm occurs normally, and in the absence of any clinical signs, this may not be indicative of any disease (47). Therefore, it is advisable to avoid routine checking of gastric residuals and abdominal girth.

## Nutrition to Preterm Infants—What to Feed?

### Growth Rate with Standard Nutritional Practices

The prevailing nutritional practices for preterm infants include minimal enteral feeds (10 mL/kg/day); use of breast milk, donor milk (if maternal milk is not available), or fortified human milk; feed advancements of 20 mL/kg/day; and parenteral nutrition with amino acids and lipids (48). Despite following standard practices, the growth in preterm infants may not be optimal in most cases. Current nutritional strategies or practices being followed for intrauterine growth restriction (IUGR)/preterm infants are not able to prevent postnatal growth restriction (49). Extrauterine growth restriction (EUGR) is a serious issue in preterm LBW infants, with an incidence of about 28, 34, and 16% for weight, length, and head circumference, respectively (50). The growth rate of preterm and extremely LBW infants during hospitalization in the neonatal intensive care unit significantly impacts neurodevelopmental and growth outcomes in later life (51).

### Current Enteral Nutritional Strategies—Pros and Cons

The enteral nutrition options for preterm infants include EBM, fortified EBM, and formula milk (52).

#### Expressed Breast Milk

Breast milk should be the milk of choice for providing nutrition to preterm LBW infants, due to its several inherent advantages (Table 2) (53–69).

**Table 2:** **Advantages of feeding breast milk**.

**Overall outcomes** Better feed tolerance (53, 54). Lower risk of NEC, sepsis, and late-onset sepsis (53–55). Reduced length of hospital stay and risk of rehospitalization (56, 57).
**Microvascular outcome** Protective role in preventing retinopathy of prematurity (58).
**Cardiovascular outcomes** Lower risk of hypertension and atherosclerosis later in life (59, 60). Improved left and right ventricular end-diastolic volume index and stroke volume index, and beneficial long-term cardiovascular outcomes (61).
**Bone health** Significant increase in whole-body bone area and bone mineral content (62).
**Neurological outcomes** Improved neurological development in later years (53). Significantly higher IQ in later years, even after adjusting for maternal IQ (63–65). Better receptive language at 3 years and verbal and non-verbal IQ at 7 years. This outcome has been noted with a longer duration of feeding breast milk; each month of feeding breast milk may increase verbal IQ by 0.35 points and non-verbal IQ by 0.29 points (66). Significant improvement in white matter microstructure, which may translate to improved cognitive, behavioral, and real-world academic performance (67, 68). Significantly higher brain volume and white matter volume, which in turn correlates with significantly improved verbal IQ, especially in boys (25% increase in IQ) (69). Improved mental and psychomotor development, and behavioral scores (57).

**Consensus recommendations on the use of mother’s milk for feeding preterm infants.**

- The first choice of human milk for feeding preterm infants is expressed breast milk from the mother; the second choice is donor pasteurized human milk.
- “Donor pasteurized human milk” should be the term used for donor milk.
- A pasteurizer should be used for pasteurization of human milk; unpasteurized milk should not be used in case of donor human milk.
- Donor pasteurized human milk should be screened for human immunodeficiency virus (HIV), hepatitis C virus (HCV), hepatitis B antigen (HBsAg), venereal disease, and bacteria, using relevant tests/cultures.
- The donor mother should also be screened for HIV, HCV, HBsAg, and venereal disease within 6 months of donating milk.
- If milk banks exist locally, pooled milk may be used, provided proper consent has been obtained.
- Milk banks should liaise with agencies for pasteurization of pooled small aliquots of breast milk.
- Donor human milk may be stored at −20°C for 6 months; preterm infants should not be fed milk stored for more than 3 months.
- Although the human milk analyzer is a useful tool to analyze the nutrient content of human milk and enable subsequent fortification, it is currently being used as only a research tool and not in day-to-day clinical practice. The standard fortification method is recommended in daily clinical settings.

Although feeding human milk has several proven benefits, it is essential to determine whether it can meet the higher nutritional needs of preterm infants. The recommended enteral macro and micronutrient intakes for preterm infants according to the European Society of Pediatric Gastroenterology, Hepatology, and Nutrition (ESPGHAN) have been summarized in Table 3 (70).

**Table 3:** **Recommended enteral nutrient intakes for preterm infants**.

Nutrient	Per kg per day	Nutrient	Per kg per day
Fluid, mL	135–200	Calcium, mg	120–140
Energy, kcal	110–135	Phosphate, mg	60–90
Protein, g	3.5–4.5	Vitamin D, IU	800–1,000
Fat, g	4.8–6.6	Vitamin A, IU	1,300–3,300
Carbohydrates, g	11.6–13.2	Iron, mg	2–3

Given the high nutrient needs of preterm infants, human milk alone may not be able to comprehensively provide the preterm infants’ requirement of proteins, energy, minerals, vitamins, and trace elements (71). Furthermore, feeding preterm infants with human milk alone may result in slower growth rates and lesser increase in head circumference as compared to feeding with fortified human milk (72). According to ESPGHAN, the preferred nutrition for premature infants is fortified human milk, preferably from the infant’s own mother, or formula milk designed for preterm infants (70).

#### Fortified Human Milk

Fortifying breast milk helps in providing additional nutrients to LBW infants whose needs are not met by EBM alone (53). Fortification is based on the principle of increasing the concentration of nutrients to such levels that the infant’s needs are met with customary feeding volumes (73). Fortification may be done with any one nutrient (monocomponent) or multiple nutrients (multicomponent). Although there is a lack of evidence supporting the benefits of monocomponent fortification with carbohydrates and fats, fortification with proteins has been found to result in an increase in weight gain, linear growth, and head growth in preterm infants. However, protein-alone fortification has not been associated with long-term growth and neurodevelopmental outcomes (74, 75). While fortifying human milk, it is essential to balance the osmolality of the feed, as fortification increases the osmolality of the feed (53).

The benefits of enteral feeds rich in docosahexaenoic acid (DHA) are debatable. Dietary DHA has been reported to be an important nutrient affecting neurological development in preterm infants, in some reviews (76). Furthermore, studies report significantly improved cognitive function in preterm girls fed enteral feeds high in DHA (77). However, a few other systematic reviews indicate no benefit or harm with long-chain polyunsaturated fatty acid supplementation in preterm infants (78).

Multicomponent fortification has been associated with short-term weight gain and linear and head growth in preterm infants. However, long-term evidences are lacking (72, 79). The National Neonatology Forum of India recommends the use of multicomponent fortification to infants born at <32 weeks’ gestation, or with <1,500 g birth weight who fail to gain weight despite receiving full volumes of breast milk (up to 180–200 mL/kg/day) (80).

#### Clinical Evidences on Fortified Human Milk

Few Indian studies have reported the outcomes of feeding fortified human milk to preterm infants. In a study by Mukhopadhyay et al., in 166 preterm infants weighing ≤1,500 g, infants fed fortified EBM experienced significantly better growth in terms of weight gain, length, and head circumference when compared to infants exclusively fed human milk. This effect was more evident in infants who were small for gestational age; length and weight were significantly increased in this group, which was fed fortified EBM (81). In a study by Gathwala et al., fortification of EBM was not associated with delayed gastric emptying or feed intolerance in preterm neonates (82). In a study by Agarwal et al., there was an increase in osmolality of breast milk after the addition of human milk fortifier (HMF). However, the osmolality did not change after storage at 4°C for 6 h (83). These results are in contrast to those noted in an international study by Henriksen et al. in preterm very LBW infants fed fortified milk. The EUGR of these infants increased to 58% at hospital discharge, from 33% at birth (84). Therefore, the outcomes may not be optimal in all cases; this may be due to the low energy and protein content of the feeds (84, 85).

Standard fortification of human milk may be associated with protein deficits, as the protein content is low during fortification, assuming the availability of higher amounts of protein in human milk (86). However, it must be noted that the protein content of EBM is variable and may differ based on the duration of lactation and from sample to sample (86, 87). Therefore, standard fortification of human milk may not meet the recommended protein intake in preterm infants (88). To overcome this limitation and optimize human milk fortification, the concept of individualized fortification has been introduced.

#### Individualized Fortification

Two methods of individualized fortification have been proposed—targeted and adjustable (86).

Targeted fortification—this method involves analyzing the protein content of human milk and fortifying it to meet the infant’s nutrient requirement. A target protein intake is chosen based on predefined infant requirements. This method helps in individualized fortification of preterm infants (89).

Adjustable fortification—the protein intake in this method is periodically adjusted based on the metabolic response of the infant evaluated from the blood urea nitrogen tests. This method is more suitable for stable preterm infants. Furthermore, it is a more practical and feasible method that does not require frequent analyses of the milk (86). In comparison to the standard fortification method, the adjustable protein fortification technique has been found to result in a significant improvement in growth indices, weight gain, and head circumference in preterm very LBW infants. This improvement significantly correlated with the higher protein intake in infants fed using the adjustable regimen (90, 91).

In this context, it may be pertinent to mention that excess protein intake to promote faster postnatal growth may not necessarily be beneficial; it may result in increased blood pressure in the long term (92). Therefore, it is essential to optimize the nutrient intake based on the infants’ requirements.

**Consensus recommendations on the supplementation of protein, calories, and calcium in daily clinical practice, for the optimum growth of preterm infants.**

- HMF may be used only when the infant reaches a feed of 100 mL/kg/day. However, based on the clinical situation, appropriate amounts of HMF may be used even before the set feed limit has been achieved.
- In clinical practice, one sachet (1 g) of HMF may be used for 20 or 25 mL of expressed or donor pasteurized human milk, depending on the product guideline.
- It is advisable to monitor the calorie and protein intake of the infant to evaluate the protein requirements.
- The recommended protein intake should be 3.5–4 g/kg/day, depending on the birth weight and desired growth.
- It is important to monitor the growth velocity of the infant, along with monitoring for osteopenia of maturity.
- The calorie requirement of a preterm infant is usually met with the addition of HMF, which provides about 4 g/kg/day of protein and 3.5–4 g/kg/day of fats.
- A higher protein and higher calorie strategy will help optimize nutrition in preterm infants.
- Calculation of protein intake while adding HMF to EBM will help in the simultaneous monitoring of the requirements for carbohydrates, fats, and calories.
- There is no need to monitor and supplement additional calcium in preterm infants on total parenteral nutrition and HMF; additional supplementation of calcium may result in nephrocalcinosis.
- Serum calcium may be measured at 12–24 h after birth, followed by regular monitoring, to prevent hypercalcemia.
- Serum phosphorus and alkaline phosphatase may be measured once a week after 2 weeks of birth, to detect any osteopenia of prematurity and to take appropriate measures.

#### Side Effects of Fortification

- Earlier studies report significantly delayed gastric emptying in some preterm infants who cannot tolerate fortified milk (93). However, recent studies suggest that fortifying breast milk may not result in clinically significant feeding intolerance if the recommended concentrations of fortifier are used (94).
- The use of fortifiers containing iron may decrease the antibacterial action of preterm milk (95).
- There is no clear evidence linking human milk fortification with NEC risk (72). Hence, weighing the benefits of growth and neurodevelopment with fortification versus possible feed intolerance and NEC is at the sole discretion of the clinician.

#### Formula Milk

Formula milk contains all essential nutrients and is specifically designed to meet the requirements of LBW infants (53).

**Consensus recommendations on choosing the right preterm formula.**

- Osmolality is a key factor that needs to be taken into consideration when choosing a preterm formula.
- Preterm formula can be used in preterm infants weighing less than 1.5 kg.
- Docosahexaenoic acid (DHA) is an important part of preterm formula.

## Optimizing Growth in Preterm Infants to Lower Cardiovascular Risk

Faster weight gain in preterm infants may be associated with an increased risk of overweight and obesity; higher body fat percentage, waist circumference, serum triglycerides, and blood pressure; endothelial dysfunction; and adverse long-term cardiovascular outcomes (96–99). Therefore, monitoring and ensuring optimal weight gain is essential throughout the entire gestational spectrum, to prevent long-term complications.

## Postdischarge Nutrition of Preterm Infants

Although there is no clear evidence on the added benefit of administering a nutrient-enriched diet to preterm infants after discharge, a few reviews suggest an improvement in growth parameters with no effect on neurodevelopmental outcomes (100, 101). Studies indicate that variations in dietary nutrient intake can contribute significantly to growth deficits in preterm infants, thus highlighting the choice of appropriate nutrition in this cohort (102, 103).

The numerous benefits of mother’s milk have been discussed previously. While a few studies have reported comparable outcomes, a few others have reported better short-term growth rates in preterm infants fed human milk compared to formula milk (104, 105). A meta-analysis of 14 randomized clinical trials reveals increased short-term growth rates with fortified human milk compared to unfortified breast milk, with no increase in the risk of NEC (72). Furthermore, fortifying human milk using a human milk-based fortifier has been found to have a significantly lower risk of NEC and lower morbidity and mortality compared to bovine milk-based fortifier (106, 107).

Owing to the beneficial effects of breast milk and the paradoxical inability of preterm LBW infants to feed at the breast, it is advised to initiate expression of breast milk soon after birth. Relaxation, massaging, and warming the breasts, hand expression, and the use of low-cost pumps may be some cost-effective interventions for expressing breast milk (108). Combining pump suction, breast compression, and hand expression has been found to enhance breast milk production (109). In any case, early nutrition in preterm LBW infants should be critically optimized to have beneficial effects, both in the short term and the long term (110).

**Consensus recommendations on postdischarge nutrition.**

- The ideal discharge weight of preterm infants may be dependent on several factors, including the ability of local services to take care of the infant after discharge.
- The sucking/swallowing ability of infants should be good at discharge.
- The following signs should be monitored and immediate medical attention should be sought for: inability to feed, lethargy, temperature alterations, and mottling/cyanosis.
- Counseling should be provided for resuscitation prior to discharge.
- The first follow-up should be done within 3–7 days, followed by a weekly follow-up.
- Preterm infants should be monitored for up to a minimum of 2 years and preferably till adolescence.
- In preterm infants who are on formula milk, switching to a standard formula is recommended after they have reached their birth centile (i.e., after catch-up has been completed). There is not much evidence on the benefits of switching after an increase in weight beyond this.
- Complementary feeding may be initiated at the corrected age of 4 months. No special attention is required while initiating complementary feeding.

## Key Conclusion

- Preterm birth survivors present with higher rates of adverse developmental disabilities and health outcomes compared to their term counterparts.
- Optimal nutrition is highly essential for growth, metabolism, and immunity in preterm LBW infants; it lowers the risk of adverse morbidities in adulthood.
- Enteral feeding may be preferred to parenteral feeding due to the complications associated with the latter.
- Early and rapid initiation of enteral feeding has several advantages compared to late and slow feeding; continuous feeding may be preferred to intermittent hourly feeding.
- Early enteral feeding does not carry any additional risk of NEC in preterm infants; on the contrary, it aids in the development of the gut and reduces the risk of infections.
- EBM should be the first choice for feeding preterm infants due to its numerous inherent advantages; donor pasteurized human milk is the second choice.
- Given the high nutrient requirements of preterm infants, especially the requirements of proteins, EBM or donor milk should be fortified with HMF without increasing the osmolality of the milk.
- In routine clinical practice, standard fortification may be followed. However, the use of targeted and adjustable fortification, where possible, may help in optimal nutrient supplementation to preterm infants.
- Monitoring the growth velocity is essential, as rapid weight gain in preterm infants may be associated with future cardiovascular risk. Hence, optimal weight gain should be the target.
- Counseling and regular follow-up after discharge and monitoring of the preterm infant, preferably until adolescence, are advisable.

## Author Contributions

All the authors contributed equally to the Conceptualization, review, and finalization of the manuscript.

## Conflict of Interest Statement

RK, AS, and UV have none to declare. SB, FA, and SR are employees of Nestle Nutrition.
